# Morphology-Controlled Fabrication of Large-Scale Dendritic Silver Nanostructures for Catalysis and SERS Applications

**DOI:** 10.1186/s11671-019-2923-0

**Published:** 2019-03-12

**Authors:** Zi-Qiang Cheng, Zhi-Wen Li, Jing-Han Xu, Rui Yao, Zong-Lin Li, Shan Liang, Guang-Ling Cheng, Yan-Hong Zhou, Xin Luo, Jiang Zhong

**Affiliations:** 1 Department of Applied Physics, School of Science, East China Jiaotong University, Nanchang, 330013 People’s Republic of China; 2 Department of Physics, Hunan Normal University, Changsha, 410081 People’s Republic of China; 3 School of Chemistry and Chemical Engineering, Jiangxi Science and Technology Normal University, Nanchang, 330013 People’s Republic of China

**Keywords:** Dendritic silver nanostructures, Catalysis, Surface-enhanced Raman scattering, Electrochemical deposition

## Abstract

Highly branched metallic nanostructures, which possess a large amount of catalyst active sites and surface-enhanced Raman scattering (SERS) hot spots owing to their large surface areas, multi-level branches, corners, and edges, have shown potential in various applications including catalysis and SERS. In this study, well-defined dendritic silver (Ag) nanostructures were prepared by a facile and controllable electrochemical deposition strategy. The morphology of Ag nanostructures is controlled by regulating electrodeposition time and concentration of AgNO_3_ in the electrolyte solution. Compared to conventional Ag nanoparticle films, dendritic Ag nanostructures exhibited larger SERS enhancement ascribed to the numerous hot spots exist in the nanogaps of parallel and vertically stacked multilayer Ag dendrites. In addition, the prepared dendritic Ag nanostructures show 3.2-fold higher catalytic activity towards the reduction of 4-nitrophenol (4-NP) by NaBH_4_ than the Ag nanoparticle films. The results indicate that the dendritic Ag nanostructures represent a unique bifunctional nanostructure that serves as both efficient catalysts and excellent SERS substrates, which may be further employed as a nanoreactor for in situ investigation and real-time monitoring of catalytic reactions by SERS technique.

## Introduction

Noble metal micro/nanostructures have attracted great attention due to their potential applications in optics [1], catalysis [2–4], surface-enhanced Raman scattering (SERS) [5–7], and solar energy harvesting [8]. The physical and chemical properties of metal micro/nanostructures are mainly determined by their size, shape, and composition [9, 10]. The controlled fabrication of metal micro/nanostructures with tunable size and morphology provides great opportunities to systematically investigate their properties and practical applications. Recently, due to the progress in nanofabrication techniques, metal nanostructures with different sizes and morphologies have been successfully prepared by using various fabrication approaches [2, 9–13].

The applications based on the substrates with plasmonic nanostructures have been extensively explored [5, 7]. Most of the fabrication strategies, such as focused ion beams lithography [13], nanoimprint lithography [14], electron beam lithography [15], nanosphere lithography [16], and self-assembly [17], are used to fabricate large-scale and uniform-sized metallic nanostructure substrates. However, these fabrication strategies are still characterized by high cost, long time, and complex processes. Therefore, it is necessary to develop a simple and efficient synthesis route of large-area and shape-controlled metal micro/nanostructures. Electrochemical deposition is a simple, powerful, and convenient technique to one-step synthesize and immobilize large-area metal micro/nanostructures onto substrates simultaneously [7, 18–26]. The morphology and size of the electrodeposited metal products can be controlled by tuning the deposition conditions, such as the concentration and proportion of electrolyte solution, electrodeposition current density, and electrodeposition time. Generally, in the growth process of nanocrystals, the final morphology depends on the formation conditions departing from thermodynamic equilibrium [18, 25–29]. Electrochemistry is widely used to study morphological transitions of nanocrystals in non-equilibrium growth processes. Due to the fast nucleation and growth of nanocrystals, non-equilibrium processes are important for synthesizing interesting structures with hierarchical morphologies [18, 22–25]. Recently, electrochemical deposition methods have been used to fabricate various metal structures, including pyramids [7], flower-like mesoparticles [18], nanosheets [19], nanorods [20, 21], dendrites [22–25], and concave hexoctahedral nanocrystals [26].

In this work, dendritic fractal nanostructures on indium tin oxide (ITO) glass substrates were fabricated by a facile and controllable electrochemical deposition strategy. The shape evolution induced by the AgNO_3_ concentration, deposition time, deposition current density, and citric acid concentration were systematically investigated to reveal the influences of AgNO_3_ concentration and deposition time on final morphologies. The prepared dendritic Ag nanostructures exhibited larger SERS enhancement and catalytic activity compared to the Ag nanoparticle films prepared by magnetron sputtering method.

## Methods/Experimental

### Fabrication of Dendritic Ag Fractal Nanostructures

Dendritic Ag fractal nanostructures were prepared by an electrochemical deposition process, which is described in our previous work [18, 25]. The electrochemical deposition process was conducted with a two-electrode system consisting of a ITO glass (1.5 × 1 cm^2^, 17 Ω/square) cathode and a platinum (Pt) plate anode. ITO glasses were cleaned by ultrasonication in acetone, distilled water, and ethanol for 15 min, respectively. The distance between the two electrodes was set to be 3 cm. The electrolyte solution contained AgNO_3_ (2 g/L) aqueous solution and citric acid (40 g/L). In the electrochemical deposition process, a constant current density of 1 mA cm^−2^ was applied. After the electrodeposition process was completed, the samples were rinsed with ultrapure water for several times and then dried with high-purity flowing nitrogen. The as-electrodeposited dendritic Ag fractal nanostructure samples were then submerged into 10^−5^ M 3,3′-diethylthiatricarbocyanine iodide (DTTCI) ethanol solution for 4 h to adsorb a self-assembled monolayer of molecules. The SERS samples were carefully rinsed with ethanol to remove the weakly bound molecules and then dried under N_2_ before analysis.

### Catalytic Reaction

In a typical 4-nitrophenol (4-NP) reduction reaction, 1 mL of 4-NP (2 × 10^−5^ M) aqueous solution was mixed with 1 mL of ice-cold NaBH_4_ (6 × 10^−2^ M) aqueous solution under magnetic stirring conditions. A piece of catalyst (the obtained dendritic Ag nanostructure sample and Ag nanoparticle films) with the size of 5 × 10 mm^2^ was added into the reaction mixture. The reducing process of 4-NP was monitored by measuring the absorption spectra of the reaction solution at regular intervals.

### Characterization

The structure of the electrodeposited Ag products was characterized by using transmission electron microscope (TEM, JEOL 2010 HT) and scanning electron microscope (SEM, FEG Sirion 200) equipped with an energy-dispersive X-ray spectrometer (EDX). X-ray diffraction (XRD) measurements were performed on a Bruker D8-advance X-ray diffractometer with Cu Kα1 irradiation (*λ* = 1.5406 Å). The time-dependent absorption spectra of the reaction solution were measured using an UV-Vis spectrophotometer (TU-1810). SERS spectra were measured by using a micro-Raman spectrometer (HORIBA Jobin Yvon LabRAM HR800). The SERS samples were excited by focusing a 488-nm laser beam onto the sample through a × 50 objective.

## Results and Discussion

### Fabrication of Dendritic Ag Fractal Nanostructures and Effect of Reaction Conditions

**Fig. 1:**
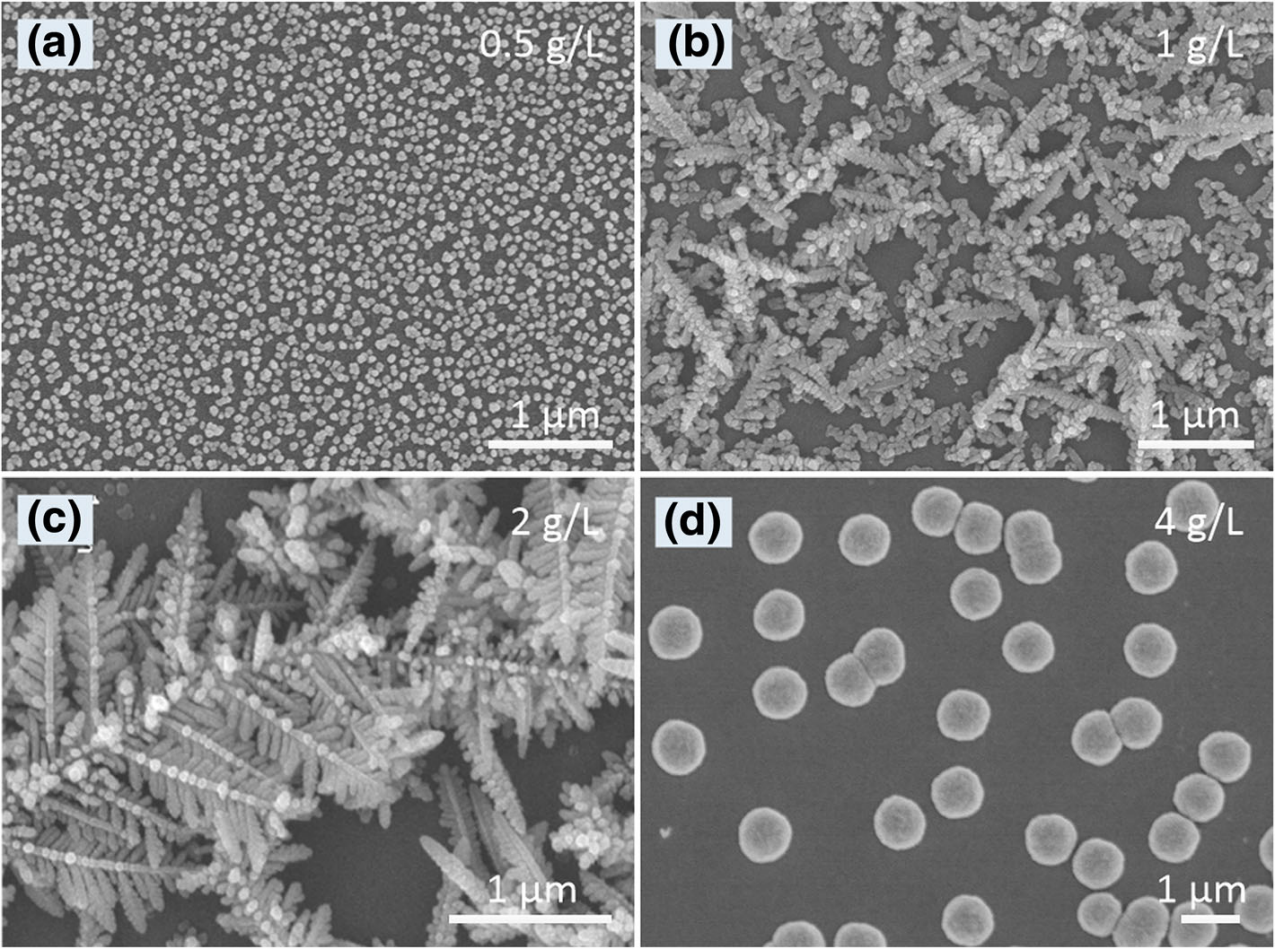
SEM images of the Ag micro/nanostructures electrodeposited under different concentrations of AgNO_3_: **a** 0.5 g/L, **b** 1 g/L, **c** 2 g/L, and **d** 4 g/L. Electrodepositing time, 90 s; the current density, 1 mA cm^−2^; 40 g/L citric acid

The electrochemical deposition method has been described as a facile and effective strategy for shape-controlled synthesis of metal micro/nanostructures due to flexible reaction conditions [7, 18, 25]. Four morphologies of Ag products (Fig. 1) were achieved by regulating AgNO_3_ concentration. Under four AgNO_3_ concentrations (0.5, 1, 2, and 4 g/L), four morphologies such as meatball-like nanoparticles (Fig. 1a), leaf-like rods (Fig. 1b), highly branched dendrites (Fig. 1c), and micro-hemispheres (Fig. 1d) were obtained. These results indicated the critical role of a proper concentration of AgNO_3_ in the formation of dendritic Ag fractal nanostructures.

**Fig. 2:**
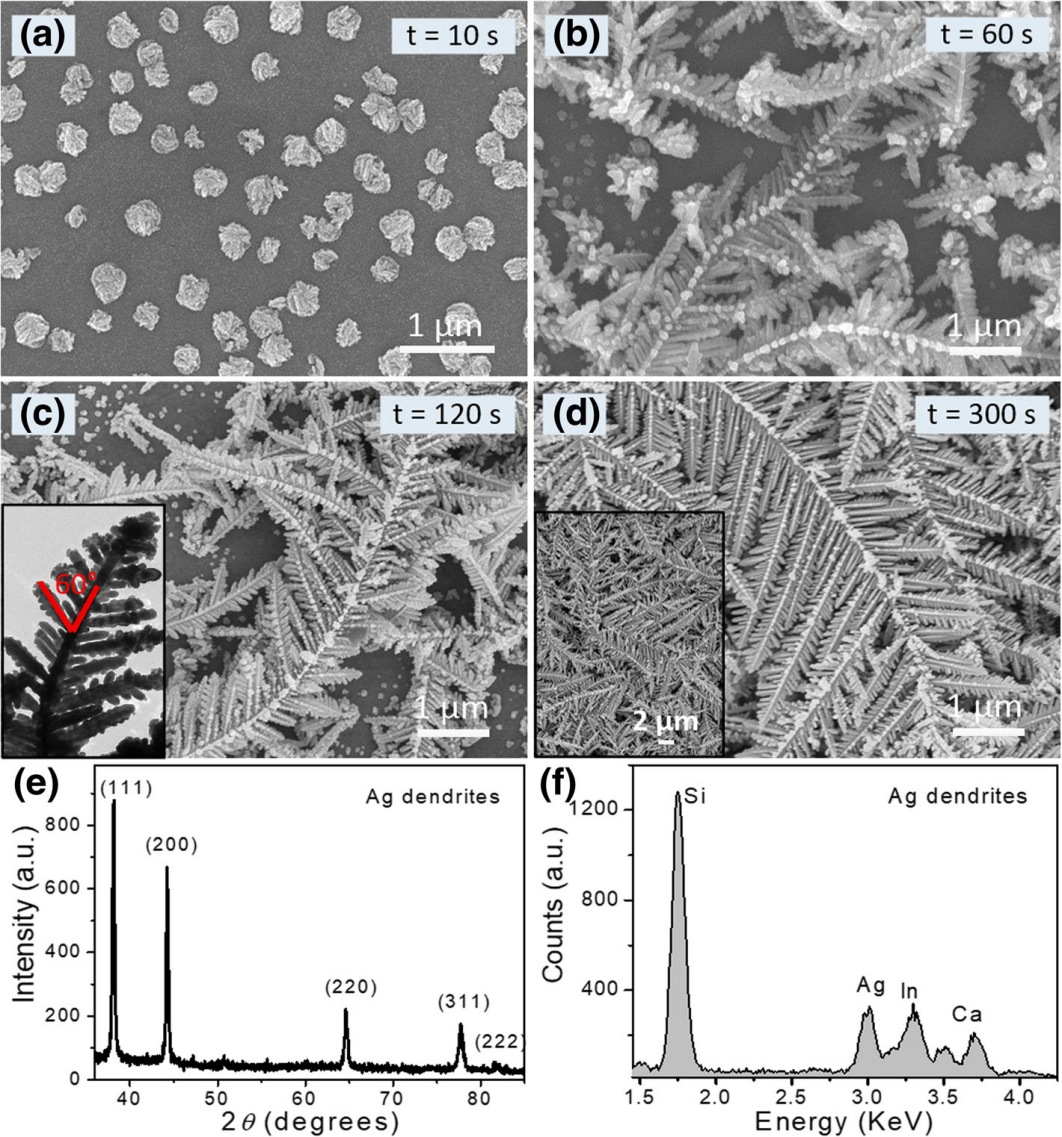
SEM images of Ag nanostructures prepared by electrodeposition for **a** 10 s, **b** 60 s, **c** 120 s, and **d** 300 s. The insets show the TEM image and low magnification SEM image of dendritic Ag nanostructures. **e** XRD pattern and **f** EDX profile of the dendritic Ag nanostructures (*t* = 300 s)

SEM images of the Ag micro/nanostructures formed after different deposition time were used to investigate the formation process of dendritic Ag fractal nanostructures. An obvious transformation stage from the flower-like nanoplate to the highly branched dendritic nanostructure during the morphological evolution was obviously identified (Fig. 2). After a short deposition time (*t* < 60 s), only some flower-like nanoplates were formed and Ag dendrites were seldom observed (Fig. 2a). When deposition time increased to 60 s, some small-branched Ag dendrites appeared at the tips of flower-like nanoplates (Fig. 2b). When deposition time increased to 120 s, bigger, longer, and more complicated Ag dendrites were formed (Fig. 2c), showing a long main trunk with secondary or multi-level branches. The branches and the central trunk displayed a selected orientation angle of c.a. 60° (inset in Fig. 2c). When deposition time further increased (*t* ≥ 300 s), the dendrites greatly extended at the lateral and vertical orientations to form a large-sized “fern-leaf” spread on the ITO glass surface (Fig. 2d). Figure 2e and f show the XRD and EDX patterns of dendritic Ag fractal nanostructures. The five diffraction peaks match well with the (111), (200), (220), (311), and (222) planes of Ag face-centered cubic (fcc) structure (JCPDS, No. 04-0783).

**Fig. 3:**
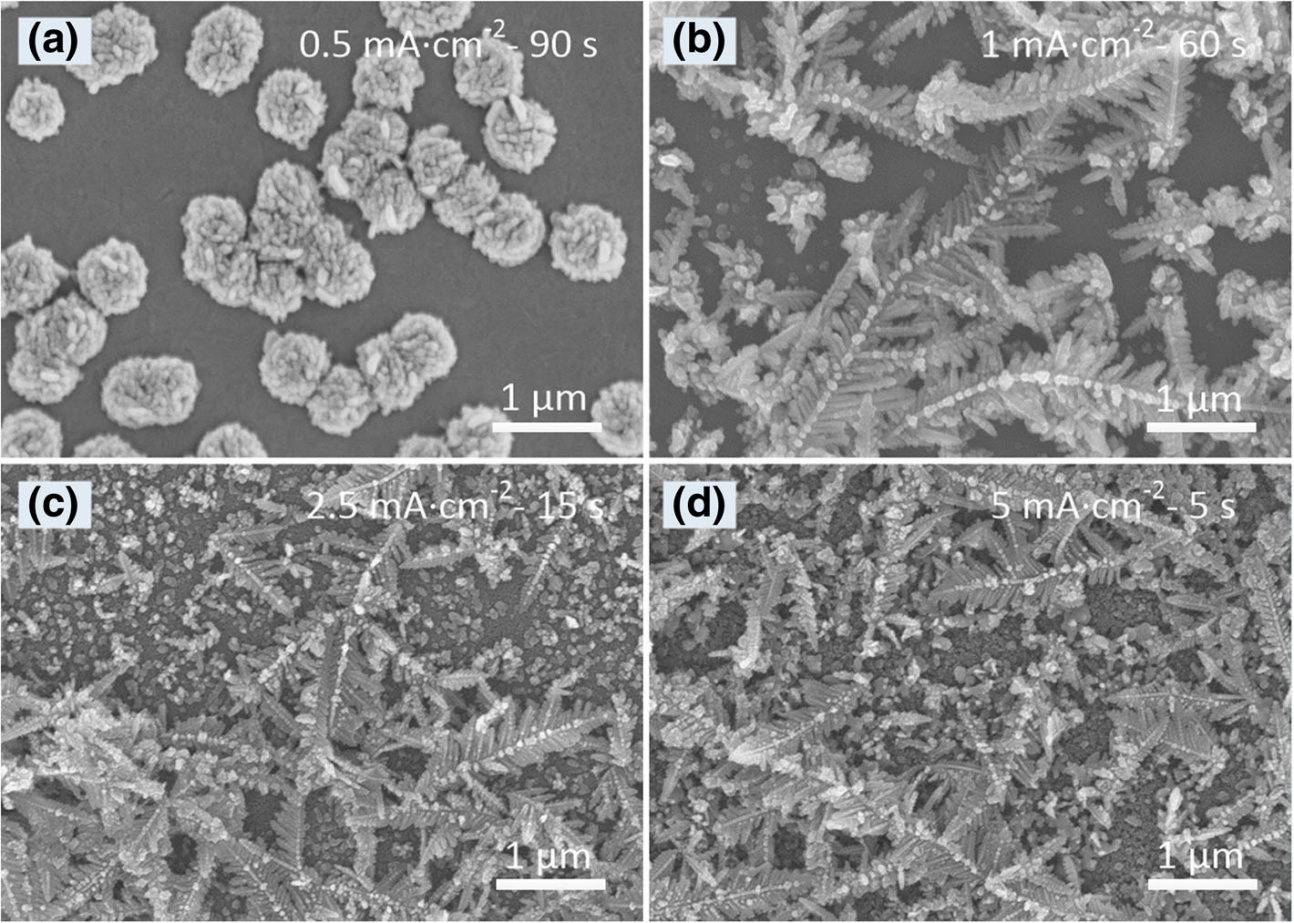
SEM images of the Ag products electrodeposited under different current densities: **a** 0.5 mA cm^−2^, **b** 1 mA cm^−2^, **c** 2.5 mA cm^−2^, and **d** 5 mA cm^−2^. The concentrations of AgNO_3_ and citric acid are 2 g/L and 40 g/L, respectively

To check the effects of current density on the morphology of electrodeposited Ag products, we changed the current density while keeping other deposition conditions unchanged (i.e., the electrolyte containing 2 g/L AgNO_3_ and 40 g/L citric acid). Under a low deposition current density (0.5 mA cm^−2^), only some micro-hemispheres grew on the ITO glass surface (Fig. 3a). When the current density was 1 mA cm^−2^, the product was mainly micro-sized Ag dendrites (Fig. 3b). When the current density was much higher (2.5 and 5 mA cm^−2^), the coexistence of Ag dendrites and nanoparticles was found on the ITO glass surface (Fig. 3c, d). High deposition current density would lead to fast growth rate. Therefore, preferential growth disappeared and more interfering species were generated on the ITO glass surface (Fig. 3c, d). Under a relatively low deposition current density, the formation and migration of Ag clusters were slow, so the newly formed Ag clusters have enough time to attach themselves onto the formed Ag dendrites and new particles would not be formed.

**Fig. 4:**
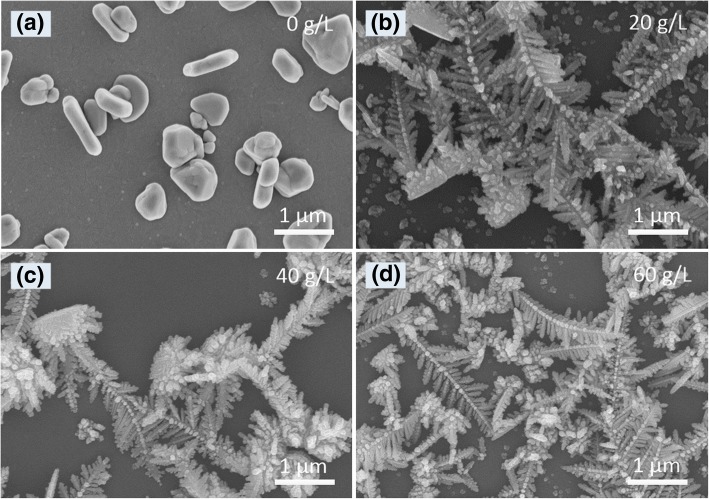
SEM images of the Ag products electrodeposited at the citric acid concentrations: **a** 0 g/L, **b** 20 g/L, **c** 40 g/L, and **d** 60 g/L. Electrodepositing time, 60 s; current density, 1 mA cm^−2^; 2 g/L AgNO_3_

The effects of citric acid concentration on electrodeposited products were also explored. Under the fixed AgNO_3_ concentration (2 g/L) and current density (1 mA cm^−2^), without citric acid in the electrolyte, only irregular micro-particles (without any dendrites) were obtained on the ITO glass surface (Fig. 4a), indicating that citric acid was a prerequisite for the formation of Ag dendrites. Ag dendrites with the uniform size and morphology could be obtained only under a medium concentration of citric acid (Fig. 4c). When the citric acid concentration was low or excessively high, Ag dendrites with different sizes and morphologies coexisted on the ITO glass surface (Fig. 4b, d).

According to the above results, the formation of dendritic Ag fractal nanostructures with the uniform size and morphology could be obtained by adjusting AgNO_3_ concentration, deposition time, deposition current density, and citric acid concentration. Obviously, the whole growth process is a non-equilibrium state as the fast nucleation and growth contribute to the formation of more complicated structures [18, 25–30]. With the departure from thermodynamic equilibrium, the diverse morphologies of final products were obtained [18, 25–30]. The diffusion-limited aggregation model can be used to interpret the non-equilibrium fractal growth process [31, 32]. In the formation process of dendritic Ag fractal nanostructures, numerous nanoparticles were firstly formed and then assembled as dendrites through oriented attachment [23, 24, 27]. The anisotropic crystal growth is ascribed to citric acid as the functional capping agent and the selective adhesion to a certain plane of Ag nanoparticles [18, 33–35].

### Catalytic Activities of Dendritic Ag Nanostructures for the Reduction of 4-Nitrophenol

**Fig. 5:**
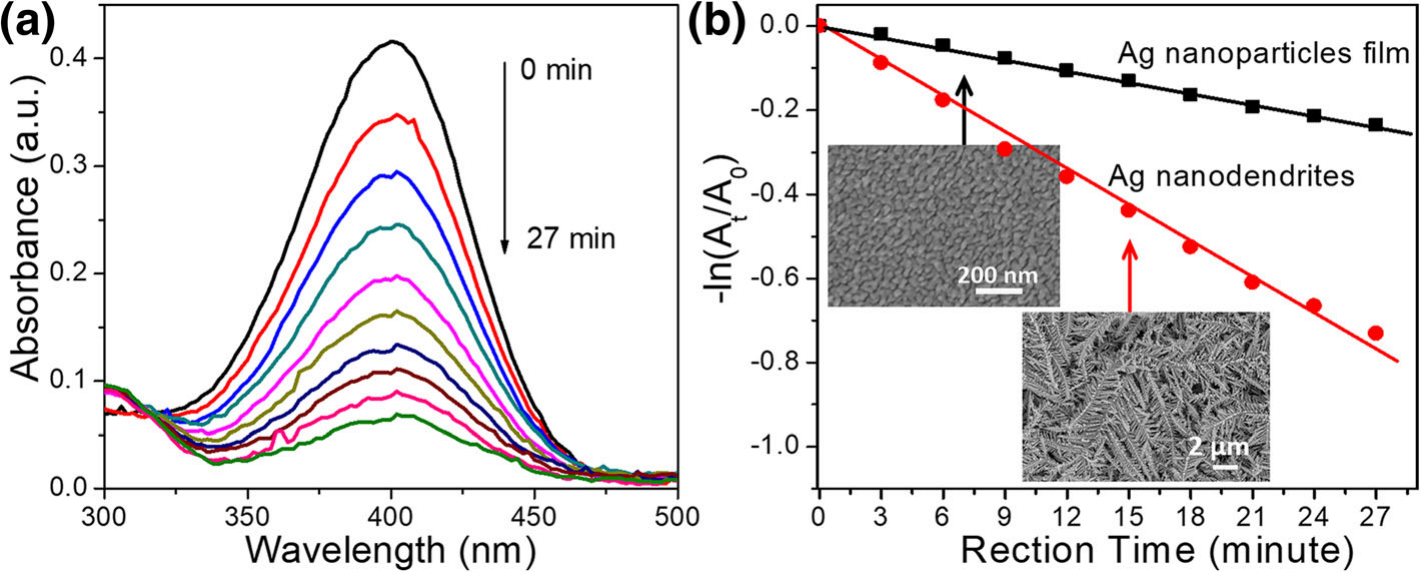
**a** Time-dependent absorption spectra of the reaction solution in the presence of the dendritic Ag nanostructures. **b** Plots of − ln [*A*_t_/*A*_0_] against time for the reduction of 4-NP catalyzed by dendritic Ag nanostructures and Ag nanoparticle film

We used the reduction reaction of 4-NP by NaBH_4_ as a model reaction to examine the catalytic activity of the dendritic Ag nanostructures. For comparison, we also explored the catalytic activity of the Ag nanoparticle film prepared by using a sputtering technique. The reaction processes were monitored by using UV-Vis spectroscopy. The time-dependent absorption spectra of the reaction solution in the presence of the dendritic Ag nanostructures are shown in Fig. 5a. The absorption peak intensity at 400 nm gradually dropped in the reduction reaction, and the shoulder at 300 nm can be ascribed to 4-aminophenol [4], the reduction product of 4-NP. The plots of − ln [*A*_t_/*A*_0_] versus time for the reduction of 4-NP catalyzed by dendritic Ag nanostructures and Ag nanoparticle film are shown in Fig. 5b. The rate constants *k* of the reaction catalyzed by dendritic Ag nanostructures, and Ag nanoparticle film were calculated to be 2.88 × 10^−2^ and 0.91 × 10^−2^ min^−1^, respectively. The reaction rate of the dendritic Ag nanostructures was about 3.2 times higher than that of the Ag nanoparticle film. The large surface area and more active sites are two rules in the design of catalysts. The dendritic Ag nanostructures exhibited the higher catalytic performance because the dendritic Ag nanostructures had a hierarchical fractal structure with large surface areas and many multi-level branches, corners, and edges, which provide a large amount of “catalyst active sites.” We thus believe that the dendritic Ag nanostructures have potential applications in catalytic reactions.

### SERS Activities of Dendritic Ag Nanostructures

**Fig. 6:**
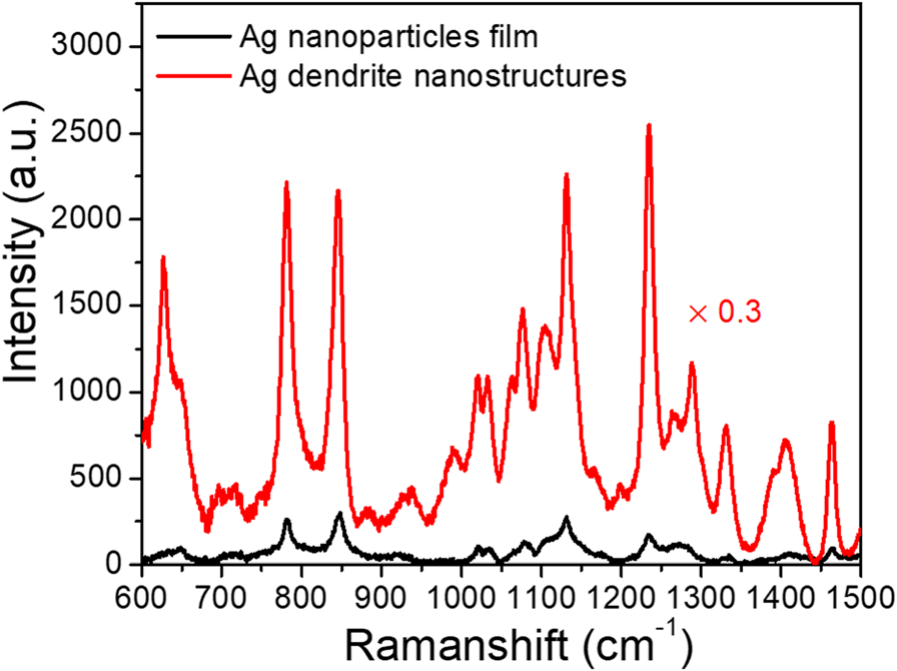
SERS spectra of 10^−5^ M DTTCI on the dendritic Ag nanostructures and the Ag nanoparticle film

Furthermore, DTTCI was chosen as the analyte molecule to investigate the SERS performance of the dendritic Ag nanostructures. Figure 6 shows the SERS spectra of the 10^−5^ M ethanol solution of DTTCI on the dendritic Ag nanostructures and the Ag nanoparticle film at 488 nm laser excitation. When DTTCI is adsorbed on the dendritic Ag nanostructures, a large Raman signal is obtained, which is attributed to the DTTCI molecules [36]. The strongest peak at 1235 cm^−1^ is utilized to compare the SERS intensity. The SERS signal of DTTCI molecules on the dendritic Ag nanostructures sample is ~ 30 times stronger than those on the Ag nanoparticle film. Ten randomly chosen spots on dendritic Ag nanostructure sample and Ag nanoparticle film were used to calculate the enhancement factor by counting the intensity ratio of SERS signal. Such large enhancement could be attributed to the fact that more hot spots with largely enhanced localized field were formed at the nanogaps of parallel and vertically stacked multilayer Ag dendrite “film.”

## Conclusion

In conclusion, we have prepared the dendritic Ag nanostructures by a facile and controllable electrochemical deposition method. AgNO_3_ concentration and electrodeposition time were the key parameters of the formation of well-defined dendritic Ag nanostructures. Dendritic Ag nanostructures exhibited larger SERS enhancement and higher catalytic activity than Ag nanoparticle films. The excellent SERS performance and high catalytic activity should be ascribed to the high-density SERS hot spots and catalyst active sites provided by the large surface area, numerous branches, tips, edges, and gaps of dendritic Ag nanostructures. This work provides a simple route for large-area and shape-controlled synthesis of dendritic Ag nanostructures as an effective catalyst and excellent SERS substrate, which may have great potential in in situ SERS investigation and monitoring of catalytic reactions.

## Abbreviations

4-NP: 4-Nitrophenol
Ag: Silver
DTTCI: 3,3′-Diethylthiatricarbocyanine iodide
EDX: Energy-dispersive X-ray spectroscopy
ITO: Indium tin oxide
SEM: Scanning electron microscope
SERS: Surface-enhanced Raman scattering
TEM: Transmission electron microscopy
XRD: X-ray powder diffraction
